# How did smokers respond to standardised cigarette packaging with new, larger health warnings in the United Kingdom during the transition period? A cross-sectional online survey

**DOI:** 10.1080/16066359.2019.1579803

**Published:** 2019-03-19

**Authors:** Crawford Moodie, Leonie S. Brose, Hyun S Lee, Emily Power, Linda Bauld

**Affiliations:** a Institute for Social Marketing, Faculty of Health Sciences and Sport, University of Stirling, Stirlingshire, Scotland, UK;; b UK Centre for Tobacco & Alcohol Studies, Nottingham, UK;; c Institute of Psychiatry, Psychology and Neuroscience, King’s College London, London, UK;; d GKT School of Medical Education, King’s College London, London, UK;; e Health Behaviour Research Centre, Cancer Research UK, London, UK;; f Usher Institute, College of Medicine and Veterinary Medicine, University of Edinburgh, Edinburgh, UK

**Keywords:** Tobacco, packaging and labelling, public policy

## Abstract

**Introduction:** In the United Kingdom, standardised packaging for cigarettes was phased in between May 2016 and May 2017. We assessed whether there was an association between using standardised packs and warning salience, thoughts about the risks of smoking, thoughts about quitting, and awareness and use of stop-smoking websites.

**Methods:** We conducted a cross-sectional online survey with current smokers aged 16 and over (*N* = 1865) recruited in two regions of England between February-April 2017, when both standardised and fully-branded packs were on the market. Participants were asked about use of standardised packs, warning salience (noticing, reading closely), and whether the packs they were using increased thoughts of the risks of smoking and quitting. They were also asked about awareness of stop-smoking websites, source of awareness (including warnings on packs), and whether they had visited a stop-smoking website.

**Results:** Most participants reported currently using standardised packs (76.4%), 9.3% were not currently using them but had previously used them, and 14.3% had never used them. Compared with never users, current users were more likely to have noticed the warnings on packs often/very often (AOR (95%CI) = 2.76 (2.10, 3.63)), read them closely often/very often (AOR(95%CI) = 2.16 (1.51, 3.10)), thought somewhat/a lot about the health risks of smoking (AOR(95%CI) = 1.92 (1.38, 2.68)), and thought somewhat/a lot about quitting (AOR(95%CI) = 1.90 (1.30, 2.77)). They were also more likely to have noticed a stop-smoking website on packs.

**Conclusions:** Consistent with the broad objectives of standardised packaging, we found that it was associated with increased warning salience and thoughts about risks and quitting.

## Introduction

The United Kingdom (UK) became the third country to fully implement standardised (or plain) tobacco packaging in May 2017, following Australia in December 2012 and France in January 2017. By September 2018, standardised packaging was mandatory in three more countries (New Zealand, Norway, Ireland), with several other countries (e.g. Hungary, Slovenia, Uruguay) due to require standardised packaging by 2020 (Canadian Cancer Society, 2018). The aim in each country to have fully implemented standardised packaging is discourage initiation, encourage quitting, help former tobacco users avoid relapse and reduce exposure to second-hand smoke (Moodie et al. undated). To date, however, very few studies outside of Australia have explored how tobacco companies, retailers or consumers respond in markets with standardised packaging.

**Figure 1.**
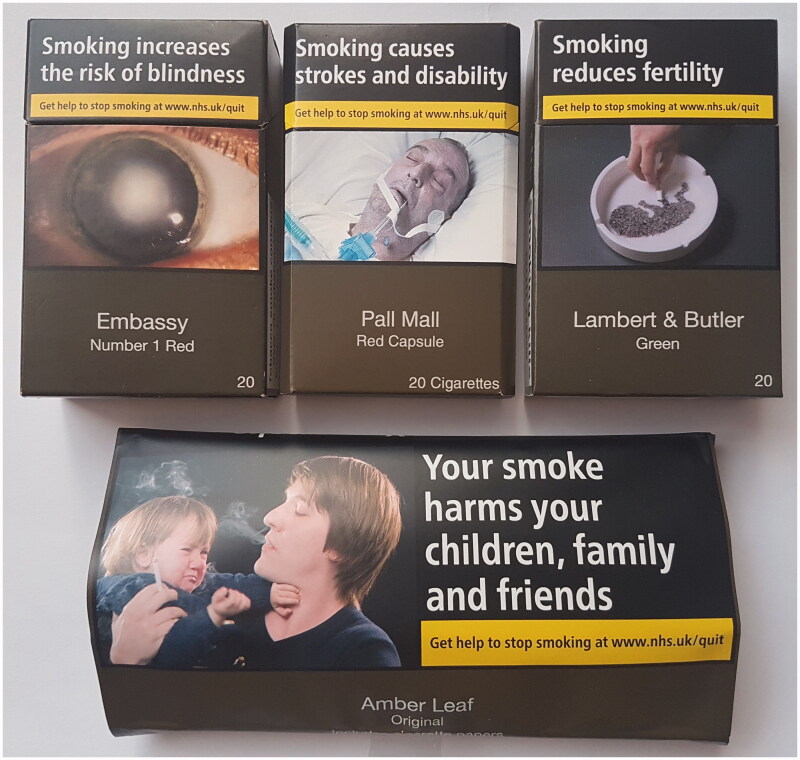
Standardised packs used in the survey.

In the UK, tobacco companies were given from 20^th^ May 2016 to 20^th^ May 2017 to implement the *Standardised Packaging of Tobacco Products Regulations* (UK Government, 2015) and also the *Tobacco Products Directive* (European Commission, 2014), which was incorporated into law through the *Tobacco and Related Products Regulations* (UK Government, 2016). The *Standardised Packaging of Tobacco Products Regulations* requires the appearance of packs of cigarettes and rolling tobacco to be standardised, including the pack colour, with the removal of all branding (colours, imagery, corporate logos and trademarks) and manufacturers only allowed to print the brand name in a mandated size, font and place on the pack. It also requires a minimum pack size of 20 for cigarettes and 30 grams for rolling tobacco, and bans any reference on the packaging to taste, smell and flavour. The *Tobacco and Related Products Regulations* (TRPR) requires pictorial warnings covering at least 65% of the principal display areas and text warnings on at least 50% of the secondary display areas. Prior to the legislation, in the UK a text warning covered 43% of the front, and a pictorial warning 53% of the back, of packs. The TRPR also requires the inclusion of cessation resource information (e.g. a stop-smoking helpline and/or web address) on each warning, with the UK Government opting to include a stop-smoking web address (Figure 1 shows standardised packaging in the UK).

Several studies in the UK have explored tobacco company and retailer response to standardised packaging. A monitor of the cigarette market, which involved a review of the trade press and online supermarkets, and regular visits to stores, found that during the first three months of the transition period tobacco companies introduced a number of limited-edition fully-branded packs and re-usable tins, and an innovative re-sealable inner foil for one brand of cigarettes (Moodie et al. 2018). As all cigarettes and rolling tobacco produced after the start of the transition period had to come in standardised packs, these findings suggest that tobacco companies had prepared to use the first few months of the transition period to continue to promote their products. A study using Electronic Point of Sale (EPoS) data from 500 small retailers was conducted to monitor the sale of the 20 top-selling cigarette and rolling tobacco brand variants (Critchlow et al. undated). None of the products monitored were sold in standardised packaging in the first five months of the transition period, and it was not until the tenth month that more products were sold in standardised packs than in fully-branded packs (Critchlow et al. in undated). A qualitative study with small retailers (*N* = 24) found that while some retailers mentioned that standardised packaging had caused some confusion, and there were occasions where customers had been given the wrong cigarettes, for many there were no problems and any issues were less common once they became familiar with the pack and name changes and as a result of stocking brands in the same positions on the gantry (display unit). Consequently, the legislation did not have much effect on transaction times and the ease of locating products on the gantry (Purves et al. undated).

In terms of compliance, a study using EPoS data from over 2400 small retailers found that ten weeks after standardised packaging became mandatory almost all (99.5%) cigarettes and rolling tobacco were sold in standardised packs (Critchlow et al. 2018). A qualitative study with small retailers similarly found compliance to be high, for three key reasons: 1) Retailers did not want to risk being fined, 2) Many had been notified by tobacco company representatives that non-compliant stock would be exchanged for free, and 3) Some retailers were aware that local wholesalers were organising events where they could swap old stock not exchanged by the tobacco company representatives (Purves et al. undated). However, there was evidence of non-compliance in both studies, with the study using EPoS data finding that 53% of the sample continued to sell a small quantity of fully-branded products ten weeks after standardised packaging became law (Critchlow et al. 2018), and one retailer in the qualitative study claiming that they were selling, and intended to continue to sell, non-compliant packs (Purves et al. undated). Some tobacco companies also appear to have failed to comply with the legislation by introducing slim standardised packs, where the width of the warnings on the secondary display areas of these packs is less than the minimum specified by the legislation (Moodie et al. 2018; Moodie et al. undated).

Few studies have explored consumer awareness of, or response to, standardised packaging in the UK. An online survey with university students (*N* = 546) between October and November 2016 found that only 11.7% had seen a standardised pack, with smokers more likely than non-smokers (17.0% vs 9.3%) to have done so (Poundall et al. 2018). That none of the leading cigarette and rolling tobacco brand variants were available in standardised packs in September 2016 (Critchlow et al. undated) helps explain why so few students had noticed any standardised packs at this time. While use of standardised packs was not assessed, most smokers reported that their likely response to the legislation would be to cut down on smoking (61%), quit (46%), switch to a cheaper brand (29%) or switch to e-cigarettes (20%) (Poundall et al. 2018). Online surveys in March 2017 with adults aged 18 and over (*N* = 2033) and youth aged 11–15 year olds (*N* = 1,041) explored whether participants had noticed any changes in tobacco packs in the previous six months. A third (32.4%) of adults reported that they had, more so current smokers (83.7%) than ex-smokers (25.1%) or never smokers (20.7%), with a fifth of youth reporting that they had noticed changes to the packaging (20.2%), more so ever smokers (49.0%) than susceptible (25.6%) and non-susceptible never smokers (16.2%) (Bogdanovica et al. 2017).

Given the dearth of research exploring consumer responses to standardised packaging in the UK, we explored the association between use of standardised packs and health warning salience, thoughts about the risks of smoking, and thoughts about quitting. Given that a stop-smoking website is mandatory on the pictorial health warnings of standardised packs for the first time across all of the UK, we also assessed awareness of stop-smoking websites, source of awareness, and whether participants had visited a stop-smoking website.

## Methods

### Design

A cross-sectional web-based survey was conducted between 27^th^ February and 21^st^ April 2017 (the last quarter of the transition period for standardised packaging), with self-reported smokers aged 16 and over in two regions of England (Yorkshire and Humber, and West Midlands), see www.picturesofengland.com/mapofengland/regions.html. These regions were selected because although the survey included questions on packaging, which is the focus of this paper, the primary aim of the study was to explore smokers’ perceptions of stop-smoking services in ‘Yorkshire and Humber’ and a region with a comparable population size. The non-probabilistic quota sample came from the online panel of YouGov, a market research company with over 810,000 panel members in the UK aged 16 and over.

### Measures

#### Demographic and smoking-related variables

Key demographic information held by YouGov include age, gender, ethnicity and social grade. Age was recorded in four strata (16–34, 35–54, 55–64, 65+) and social grade assessed using the National Readership Survey social grade classification (National Readership Survey, undated), and collapsed into ABC1 (middle and upper classes) and C2DE (working classes). Ethnicity was recorded using 2011 census categories (Office for National Statistics, 2014) and recoded into ‘white British’ and ‘other’.

Participants were asked about how frequently they smoked cigarettes (factory-made or hand-rolled), if at all. They were also asked about the number of cigarettes they typically smoke per day (daily smokers) or per week (weekly smokers), and time to first cigarette (TTFC) on the days that they smoke. TTFC and daily cigarette consumption (which was calculated for weekly smokers) were combined to give a score on the Heaviness of Smoking Index (HSI), ranging from 0–6 (Kozlowski et al. 1994). Participants were asked about past quit attempts: ‘How many attempts, if any, have you made to quit smoking in the past 12 months? Please include any attempts you’re currently making’ with the response options (No attempts; 1 attempt; 2 attempts; 3 or more attempts; Not sure, but at least one; Don’t Know) dichotomised into ‘None/Don’t know’ and ‘At least one’. Participants were asked about on-going attempts to reduce consumption or quit: ‘Are you currently trying to cut down or quit smoking?’ with the response options (Yes, trying to cut down; Yes, trying to quit; No; Not sure) dichotomised into ‘No/Not sure’ and ‘Yes’.

#### Standardised packaging use

Participants were shown an image of standardised packs, see Figure 1, and asked ‘Does the pack that you are currently using look like the ones shown in the image, i.e. with a greenish-brown colour, the brand name at the bottom, and picture warnings on the front and back?’ with response options ‘Yes’, ‘No’ and ‘Not sure’. Those who answered ‘No’ or ‘Not sure’ were asked ‘Have you previously used a pack that looks like the ones shown in the image?’ with response options ‘Yes, once or twice’, ‘Yes, several times’, ‘Yes, many times’, ‘No’ and ‘Not sure’. Responses were collapsed into Current users, Previous users and Never users.

#### Salience of health warnings

Warning salience was assessed with ‘In the last month how often, if at all, have you noticed the warning labels on packs?’ and ‘In the last month how often, if at all, have you read or looked closely at the warning labels on packs?’ For both questions, response options (Very often; Often; Sometimes; Rarely; Never; Don’t know) were dichotomised into ‘Very often/often’ and ‘Sometimes or less/Don’t know’.

#### Thoughts about health risks and quitting

Thoughts about health risks was assessed with ‘To what extent, if at all, does the look of the pack you are currently using make you think about the health risks of smoking?’ Thoughts about quitting was assessed with ‘To what extent, if at all, does the look of the pack you are currently using make you more likely to think about quitting smoking?’ For both questions, response options (Not at all; A little; Somewhat; A lot; Don’t know) were collapsed into ‘A lot/somewhat’ and ‘A little or less/Don’t know’.

#### Awareness of, and engagement with, stop-smoking websites

Participants were asked ‘In the last month, have you noticed any information or adverts about a stop-smoking website?’ (Yes, No, Not sure). Those responding ‘Yes’ were subsequently asked ‘Where did you notice information or adverts about a stop-smoking website?’ and to check all that apply for the following response options: a) Warnings on packs of cigarettes or rolling tobacco; b) TV; c) Radio; d) Newspapers or magazines; e) Posters or billboards; f) Brochure, newsletter or flyer; g) At a bus stop or on a bus; h) In the workplace; i) On the internet; j) Social media e.g. Facebook, Twitter; k) GP surgery; l) Other; m) Don’t know. They were then asked ‘In the last month, have you visited a stop-smoking website to get advice about quitting?’ (Yes, No, Can’t remember).

### Sample and procedure

The inclusion criteria were that participants were at least weekly smokers of cigarettes (factory-made or hand-rolled). Our target sample of 2,000 cigarette smokers was based on practical (cost) considerations. While response rate details are not available when using this sampling methodology, the completion rate was 36% and the achieved sample 2,034 participants. Data were weighted by age, gender and social grade to be representative of both regions with information on age and gender taken from the Office for National Statistics mid-year population estimate 2015 (Office for National Statistics, 2016) and social grade from the National Readership Survey 2016 (National Readership Survey, undated). Where information on social grade (*n* = 18), ethnicity (*n* = 4) or time to first cigarette (*n* = 33) was missing, or responses to open-ended questions were nonsensical (*n* = 1), participants were excluded. Participants that were ‘Not sure’ about whether they were currently using, or had previously used, standardised packs (*n* = 113), were also excluded, leaving 1,865 fully completed responses.

YouGov employs an active sampling method, which means that only those members of their panel that are invited to participate can do so. A sub-sample is drawn from their panel that is intended to be representative of the target sample. YouGov sent an e-mail invitation to randomly selected panel members within the two regions of England selected to participate in this survey, with a link to do so. For those who clicked on the survey link, prior to answering any questions they were given information on confidentiality, anonymity and the right to withdraw at any time, and required to provide consent. As with previous research, participants were credited with 50 points (equivalent to 50p) to their YouGov account once the survey was completed (Hooper et al. 2017). Ethical approval was obtained from the King’s College London Research Office (LRS-16/17-4373).

### Analysis

Statistical analysis was conducted using SAS 9.4M5. All analyses were run on weighted data. Chi-square tests were used to compare demographic and smoking-related characteristics by standardised packaging use (current users, previous users, never users). Bivariate and multivariable logistic regressions were used to assess the association between standardised packaging use and i) noticing health warnings; ii) closely reading the warnings; iii) thinking about risks of smoking; and, iv) thinking about quitting. The multivariable analysis was adjusted for age, gender, social grade, daily/non-daily smoking status, HSI (continuous), past quit attempts and current attempts to cut down or quit. With more than 90% of the sample white British, ethnicity was not included in the regression analysis. While bivariate and multivariable analyses were planned for awareness and use of stop-smoking websites this was not possible given that only a small number of previous and never users of standardised packs had noticed information about stop-smoking websites, so only frequencies are presented.

### Results

#### Sample characteristics

The sample was evenly split by gender, with more participants from lower social grades (56.7%) and the greatest proportion in the 35–54 year old range (45.7%). The vast majority of participants identified as white British (90.6%). The sample was predominantly daily smokers (85.3%), with approximately two-fifths (58.6%) having made at least one quit attempt in the past year and just over half (56.4%) currently trying to quit or reduce smoking (see Table 1).

**Table 1.** Sample characteristics and use of standardised packaging (unweighted *N* = 1865).

	Weighted total sample	Current users (unweighted n = 1428)	Previous users (unweighted n = 164)	Never users (unweighted n = 273)	Comparison
Age, %					
16–34	24.9%	24.9%	29.8%	21.5%	*χ*2 = 14.53, *p* = .024
35–54	45.7%	46.1%	48.0%	41.8%	
55–64	16.8%	16.2%	15.8%	20.7%	
65+	12.6%	12.7%	6.5%	16.0%	
Gender, n (%)					
Male	50.3%	48.2%	57.8%	56.4%	*χ*2 = 10.46, *p* = .005
Female	49.7%	51.8%	42.2%	43.6%	
Social Grade, n (%)					
ABC1	43.3%	43.7%	42.6%	41.7%	*χ*2 = 0.42, *p* = .81
C2DE	56.7%	56.3%	57.4%	58.3%	
Ethnicity, n (%)					
British White	90.6%	91.5%	89.8%	86.0%	*χ*2 = 8.09, *p* = .018
Other	9.4%	8.5%	10.2%	14.0%	
Quit attempts in past 12 months, n (%)					
None / DK	58.6%	57.5%	55.5%	66.4%	*χ*2 = 8.10, *p* = .017
At least one	41.4%	42.5%	44.5%	33.6%	
Smoking, n (%)					
Non-daily	14.7%	13.9%	18.2%	16.8%	*χ*2 = 3.46, *p* = .18
Daily	85.3%	86.1%	81.8%	83.2%	
Currently trying to quit / reduce consumption, n (%)					
No / DK	43.6%	42.5%	41.5%	50.9%	*χ*2 = 6.80, *p* = .03
Yes	56.4%	57.5%	58.5%	49.1%	
Heaviness of Smoking Index					
Mean (SD)	2.34 (1.49)	2.34 (1.48)	2.49 (1.49)	2.25 (1.58)	*F* = 1.41, *p* = .25

#### Standardised packaging use

Most of the sample (76.4%) reported being current users of standardised packaging, with 9.3% previous users and 14.3% never users. Of those who had previously used, but were not currently using a standardised pack, 47.0% had used one once or twice, 37.3% several times, and 15.7% many times. The groups differed in age (previous users were less likely to be aged over 64 years old), gender (current users were more likely to be females), and whether they had made any quit attempts in the last 12 months (never users were least likely to have made a quit attempt in the past 12 months), see Table 1.

#### Salience of health warnings

**Figure 2.**
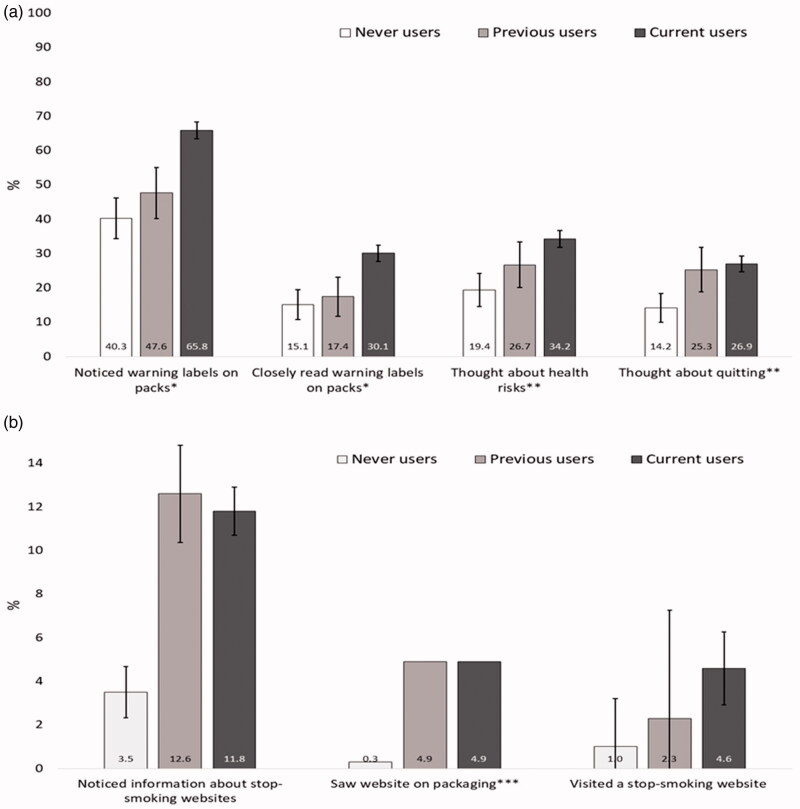
Standardised packaging and (a) salience of health warnings, thoughts about health risks and quitting; (b) awareness of and engagement with stop-smoking websites.

Three-fifths (60.5%) had often or very often noticed warnings on packs in the last month, with current users of standardised packs more likely than never users to have noticed warnings (OR  =  2.86, 95% CI: 2.19 to 3.74, *p* < .001); this association remained when adjusted for demographic and smoking characteristics (adjusted OR  =  2.76, 95% CI: 2.10 to 3.63, *p* < .001; Figure 2 and Table 2). Approximately a quarter (26.8%) had often or very often read or looked closely at warnings on packs, with current users more likely than never users to have done so (OR  =  2.43, 95% CI: 1.70 to 3.46, *p* < .001); this association remained when adjusted for demographic and smoking characteristics (adjusted OR  =  2.16, 95% CI: 1.51 to 3.10, *p* < .001; Figure 2 and Table 2). Previous users were not significantly more likely than never users to have noticed (OR  =  1.35, 95% CI: 0.92 to 1.98, *p* = 0.13; adjusted OR  =  1.31, 95% CI: 0.88 to 1.95, *p* = 0.18) and read warnings (OR  =  1.19, 95% CI: 0.71 to 1.99, *p* = 0.51; adjusted OR  =  1.00, 95% CI: 0.59 to 1.69, *p* < .001; Figure 2 and Table 2).

**Table 2.** Association between standardised packaging use and salience of health warnings (*N* = 1865).

	Noticed warning labels on packs (Very often/often)^+^				Closely read warning labels on packs (Very often/often)^+^			
	%	AOR	95% CI	p-value	%	AOR	95% CI	p-value
Standardised packaging								
Never users	40.3%	1			15.1%	1		
Current users	65.8%	2.76	2.10,3.63	**<0.001**	30.1%	2.16	1.51,3.10	**<0.001**
Previous users	47.6%	1.31	0.88,1.95	0.18	17.4%	1.00	0.59,1.69	1.00
Age								
16–34	68.1%	1			36.6%	1		
35–54	57.0%	0.67	0.52,0.86	**0.001**	26.4%	0.67	0.52,0.86	**0.002**
55–64	57.7%	0.76	0.56,1.05	0.10	19.3%	0.48	0.34,0.68	**<0.001**
65+	61.8%	0.88	0.62,1.24	0.47	18.8%	0.46	0.31,0.68	**<0.001**
Gender								
Male	55.1%	1			21.5%	1		
Female	65.9%	1.44	1.18,1.75	**<0.001**	32.1%	1.46	1.18,1.82	**<0.001**
Social Grade								
ABC1	60.7%	1			25.4%	1		
C2DE	60.4%	0.98	0.80,1.20	0.84	27.8%	1.05	0.84,1.31	0.68
Quit attempts in past 12 months								
None / Don’t know	55.9%	1			21.8%	1		
At least one	67.0%	1.46	1.18,1.81	**<0.001**	33.8%	1.51	1.20,1.90	**<0.001**
Heaviness of Smoking Index		0.94	0.87,1.00	0.05		0.97	0.90,1.04	0.38
Currently trying to quit / reduce								
No / Don't know	55.8%	1			20.9%	1		
Yes	64.2%	1.16	0.94,1.43	0.16	31.3%	1.41	1.11,1.78	**0.004**

+ Weighted data; Odds ratios and Confidence Intervals adjusted for age, gender, social grade, quit attempts in past 12 months, Heaviness of Smoking Index and Currently trying to quit/reduce.

Bold values indicate statistically significant findings.

#### Thoughts about health risks and quitting

Almost a third (31.4%) reported that the look of their pack had made them think somewhat or a lot about the health risks of smoking (Figure 2). Compared with never users of standardised packs, current users were more likely to have thought about the health risks of smoking (OR  =  2.16, 95% CI: 1.57 to 2.99, *p* < .001); this association remained when adjusted for demographic and smoking characteristics (adjusted OR  =  1.92, 95% CI: 1.38 to 2.68, *p* < .001). While previous users were more likely than never users to have thought about the risks of smoking (OR  =  1.51, 95% CI: 0.96 to 2.37, *p* = 0.07; adjusted OR  =  1.25, 95% CI: 0.78 to 1.99, *p* = 0.36), these differences were not statistically significant (Table 3). A quarter (25.0%) reported that the pack made them think somewhat or a lot about quitting (Figure 2). Compared with never users, previous users (OR  =  2.05, 95% CI: 1.26 to 3.33 *p* = 0.004; adjusted OR  =  1.90, 95% CI: 1.30 to 2.77, *p* < .001) and current users (OR  =  2.22, 95% CI: 1.55 to 3.20, *p* < .001) were more likely to have thought about quitting; for current users, this association was attenuated after adjusting for demographic and smoking characteristics (adjusted OR  =  1.64, 95% CI: 0.99 to 2.71, *p* = 0.06; Table 3).

**Table 3.** Association between standardised packaging use and thoughts about health risks and thoughts about quitting (*N* = 1865).

	Thought about health risks (A lot/somewhat)^+^				Thought about quitting (A lot/somewhat)^+^			
	%	AOR	95% CI	p-value	%	AOR	95% CI	p-value
Standardised packaging								
Never users	19.4%	1			14.2%	1		
Current users	34.2%	1.92	1.38,2.68	**<0.001**	26.9%	1.90	1.30,2.77	**<0.001**
Previous users	26.7%	1.25	0.78,1.99	0.36	25.3%	1.64	0.99,2.71	0.06
Age								
16–34	41.5%	1			32.2%	1		
35–54	31.3%	0.69	0.54,0.88	**0.003**	26.3%	0.81	0.62,1.05	0.11
55–64	23.3%	0.48	0.34,0.68	**<0.001**	17.1%	0.47	0.32,0.69	**<0.001**
65+	22.7%	0.48	0.33,0.70	**<0.001**	16.2%	0.48	0.31,0.73	**<0.001**
Gender								
Male	27.6%	1			22.0%	1		
Female	35.2%	1.17	0.95,1.44	0.14	27.9%	1.10	0.88,1.38	0.39
Social Grade								
ABC1	27.5%	1			21.2%	1		
C2DE	34.4%	1.34	1.09,1.67	**0.01**	27.8%	1.36	1.08,1.72	**0.01**
Quit attempts in past 12 months								
None / Don’t know	23.9%	1			17.2%	1		
At least one	41.9%	1.73	1.39,2.15	**<0.001**	36.0%	1.87	1.48,2.36	**<0.001**
Heaviness of Smoking Index		0.95	0.88,1.02	0.16		0.99	0.91,1.07	0.79
Currently trying to quit / reduce								
No / Don't know	21.3%	1			13.4%	1		
Yes	39.2%	1.89	1.51,2.38	**<0.001**	33.9%	2.62	2.03,3.38	**<0.001**

+ Weighted data; Odds ratios and Confidence Intervals adjusted for age, gender, social grade, quit attempts in past 12 months, Heaviness of Smoking Index and Currently trying to quit/reduce.

Bold values indicate statistically significant findings.

#### Awareness of, and engagement with, stop-smoking websites

Overall, 10.7% noticed information or adverts about stop-smoking websites in the last month (Figure 2), with the most common sources of awareness among those participants being General Practitioner surgeries (47.7%), warnings on packs of cigarettes or rolling tobacco (40.1%), television (38.5%), online (35.2%), posters/billboards (32.5%), social media (23.5%), at a bus stop/on a bus (19.2%), radio (14.8%), newspapers/magazines (12.4%), and flyers/brochures (11.9%). Only 3.9% reported having visited a stop-smoking website. Awareness of, and engagement with, stop-smoking websites was particularly low among never users of standardised packaging (Figure 2), which precluded statistical comparison between groups.

## Discussion

We found that smokers in the UK currently using standardised packs were more likely than those who had never used standardised packs to have noticed and read or looked closely at the health warnings, thought about the risks, and thought about quitting due to the look of the pack. They were also more likely to report awareness of a stop-smoking website and cite warnings on packs of cigarettes or rolling tobacco as a source of awareness.

That the health warnings used on standardised packs were novel, larger than those used on fully-branded packs, and displayed pictorial images on both main display areas (rather than just the pack reverse), may help to explain these findings, particularly in relation to warning salience and thoughts about the health risks. As such, and is the case with research on standardised packaging in Australia (e.g. White et al. 2015; Yong et al. 2016), it is not clear whether the findings are a result of the new on-pack warnings, the removal of full branding, or both.

With respect to thoughts about cessation, we found that both previous and current users of standardised packs were more likely to have thought about quitting than those who had never used these packs. This extends an online survey conducted six months into the transition period, which found that almost half of smokers (46%) thought that their likely response to the legislation would be to quit (Poundall et al. 2018). It is also consistent with the only study in Australia to have explored smokers’ responses to standardised packaging during the phase-in period, when both standardised and fully-branded packs were on sale (Wakefield et al. 2013). In a cross-sectional telephone survey with smokers (*N* = 536), it was found that those using a standardised pack were more likely than those using a fully-branded pack to have thought about quitting at least once a day in the past week and to rate quitting as a higher priority (Wakefield et al. 2013). While experimental research has shown that standardised packaging can strengthen the impact of large health warnings (e.g. Andrews et al. 2016; Harris et al. 2018), as our question about thoughts of quitting specifically asks about the look of the pack it is not possible to separate the impact of the warnings from the removal of full branding. Researchers in Australia similarly concluded that their findings must be considered the result of all the changes to the packaging (White et al. 2015), consistent with the view of marketers, that the ‘overall effect of the package comes not from any individual element but rather from the gestalt of all elements working together as a holistic design’ (Orth and Malkewitz 2008).

The inclusion of cessation resource information on warnings may motivate people to seek help and provides the opportunity to link those interested in quitting smoking with resources to help them do so (Noar et al. 2016). We found that one in ten participants reported noticing information or advertising about a stop-smoking website in the last month, with two-fifths (40.1%) of those noticing this information on packs. That more participants were aware of this information from the on-pack warnings than from all other sources (including TV, internet, radio, print and social media), except for doctor’s surgeries, highlights the value of the pack as a means of signalling available help. As only three-quarters of the sample were currently using packs that display this information (i.e. standardised packs), then awareness of the stop-smoking web address on packs among smokers will likely have increased post-standardised packaging. The warnings in the UK do not also include a quitline number however, as recommended by Article 14 of the Framework Convention on Tobacco Control (World Health Organisation, 2010), and this may prevent smokers from having an easily accessible number to hand when contemplating a quit attempt (Pierce et al. 2012; Noar et al. 2016). Of the first five countries to fully implement standardised packaging only the UK (and Norway) failed to include both a stop-smoking web address and quitline number (Moodie et al. undated). This is a missed opportunity given that not all smokers will necessarily want to, or be in a position to, access a stop-smoking website; approximately 10% of adults in the UK have never used the internet (Office for National Statistics, 2017). In addition, a systematic review and meta-analysis of internet interventions for smoking cessation found that in terms of abstinence from smoking there were no statistically significant differences in comparison with counselling delivered via telephone interventions (Graham et al. 2016).

In terms of limitations, the sample was recruited from two regions of England and while there is no reason to expect different responses in other regions of the UK, the study provides no insight into the response of smokers from across the rest of the UK. The use of an online panel also means that the findings may not be generalisable to the wider smoking population. While smokers using standardised packs were more likely to have thought about quitting due to the look of the pack, our cross-sectional design does not allow us to explore whether this resulted in any quit attempts. In addition, the findings may have been influenced by the novelty of standardised packaging, which only became more widely available for the leading tobacco brands towards the end of the transition period in the UK (Critchlow et al. undated; Purves et al. undated). While our intention was to explore any differences in warning salience, risk perceptions, and thoughts about quitting, based upon use of standardised packaging (current, previous, never), we did not ask those currently using standardised packs when they started using them. This would have allowed us to explore whether there was a dose-response effect. Future research during the transition period of standardised packaging in other markets could explore this and reasons behind previous use, e.g. are these individuals more likely to have switched from their usual brand because it was only available in standardised packs?

The UK Department of Health estimates that standardised packaging will have a net benefit to government of £25 billion ten years post-implementation (Department of Health, 2015). It is critical that countries robustly evaluate the impacts of this measure (Vardavas et al. 2017) and do so over the longer term; a major limitation identified in a Cochrane review was the absence of research exploring the longer-term impacts of standardised packaging (McNeill et al. 2017). While our findings provide support for standardised packaging during the transition period, research is needed to explore the intended and any unintended consequences of this policy in the UK (and elsewhere) after it has been fully implemented.
